# Evaluation of Flavonoids and Furanocoumarins from *Citrus bergamia* (Bergamot) Juice and Identification of New Compounds

**DOI:** 10.3390/molecules13092220

**Published:** 2008-09-18

**Authors:** Claudio Gardana, Federico Nalin, Paolo Simonetti

**Affiliations:** 1 DiSTAM - Department of Food Science and Microbiology, Division of Human Nutrition, University of Milan, 20133 Milan, Italy; E-mail: paolo.simonetti@unimi.it (Paolo Simonetti); 2 Specchiasol s.r.l., Via Rizzi 1/3 - 37012 Bussolengo (Vr), Italy; E-mail: federico.nalin@specchiasol.it (Federico Nalin)

**Keywords:** Flavonoids, Furanocoumarins, Bergamot juice, LC, Mass spectrometry

## Abstract

Bergamot juice (BJ) contains different classes of flavonoids (e.g. flavanones and flavones) that can exert beneficial effects on human health. The aim of this study was to evaluate the qualitative and quantitative composition of a BJ obtained from fruits harvested in Southern Italy (Calabria) at the end of their maturation period. The identity of several flavonoids and furanocoumarins was assessed by co-chromatography, UV spectra and molecular weight comparison. The unknown compounds were dissociated by induced collision (CID-MS) and their identity established through the characteristic ions product. By this approach a complete profile of about twenty compounds (furano-coumarins, flavonoids C- and O-glycosides) present in BJ was obtained. Furthermore, three acylated flavanones, present in amounts of 20.1±1.1, 89.3±2.2 and 190.1±3.1 mg/L, respectively, and which seem to correspond to di-oxalate derivatives of neoeriocitrin, naringin and neohesperidin, were identified for the first time in BJ. The other main flavanones were naringin, neohesperidin and neoeriocitrin, and their content was 167.5±1.8, 123.9±1.7 and 73.3±1.6 mg/L, respectively. Concerning flavones, the total amount in BJ was about 160 mg/L and the main ones were vicenin-2, stellarin-2, rhoifolin and neodiosmin. Bergapten and bergamottin were the primary furanocoumarins in BJ and their amounts were 9.0±0.4 and 18.2±0.5 mg/L, respectively.

## Introduction

Flavanones represent a small group of compounds present in high concentrations in different citrus like orange, grapefruit and lemon and in lesser amounts also in tomatoes, some aromatic plants and propolis. In citrus juice the main aglycones are naringenin, hesperetin and eriodictyol glycosylated with neohesperidose, which imparts a bitter taste, or rutinose that is flavourless. Bergamot (*Citrus bergamia* Risso) fruit and juice (BJ) are a source of bitter taste compounds containing mainly the flavanones neohesperidin, naringin, neoeriocitrin and lesser amounts of flavones and furanocoumarins. After ingestion as food the flavanone glycosides are metabolised by human intestinal bacterial microflora to the respective aglycones [1, 2], which seem to possess antioxidant [3], anticarcinogenic [4], hepatolipidemic [5] and anti-inflammatory [6] activities. In addition, naringenin and hesperetin can bind to estrogen receptors [7], or inhibit the activity of aromatase, the rate-limiting enzyme in the conversion of androgens to estrogens [8]. Naringin was found to lower total cholesterol and low-density lipoprotein cholesterol levels in plasma [9], and hesperidin to also significantly lower plasma triglyceride levels [10]. Chronic administration of BJ caused in rats a significant reduction in serum cholesterol, triglycerides, LDL and an increase in HDL levels [11]. Moreover, the authors observed a protective effect on the hepatic parenchyma. Bergamot juice contains diverse compounds with similar chemical structure thus a sophisticated high resolution technique is necessary for their identification and quantification. For this reason liquid chromatography coupled to DAD and mass spectrometry is the best choice for the analytical characterization of citrus [12,13,14,15]. The aim of this study was (1) to develop a LC-DAD-ESI-MS(MS) method allowing the qualitative and quantitative evaluation of flavonoids and furanocoumarins and (2) to identify unknown polyphenol compounds in the bergamot juice by LC-MS^2^. 

## Results and Discussion

**Figure 1:**
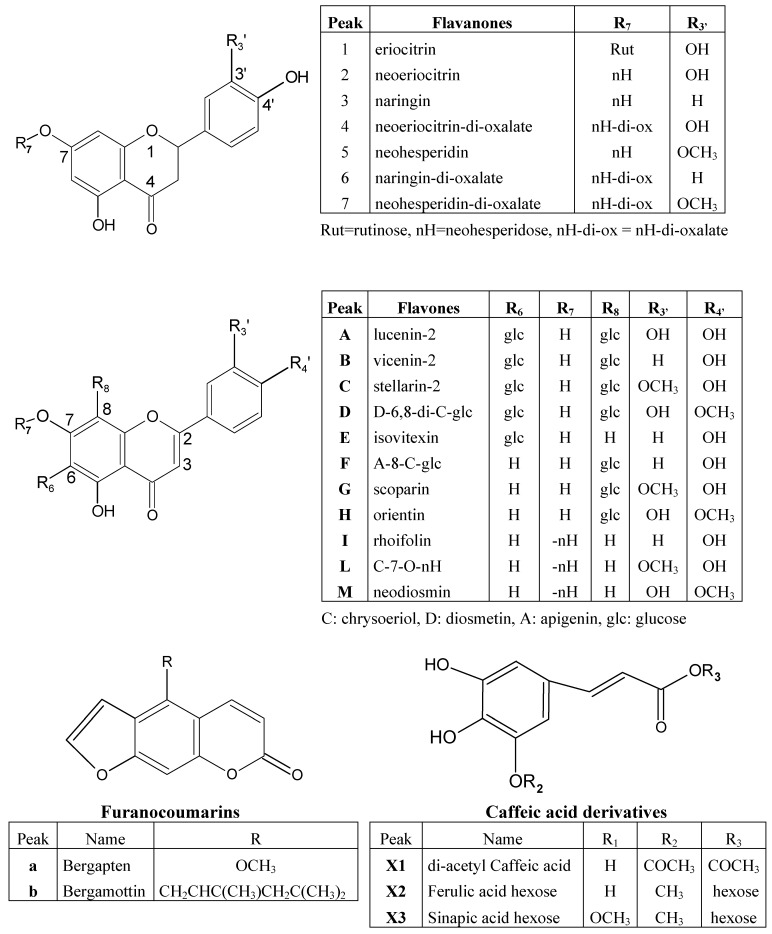
Structures of flavonoids, cinnamic acid derivatives and furanocoumarins founds in BJ.

**Figure 2:**
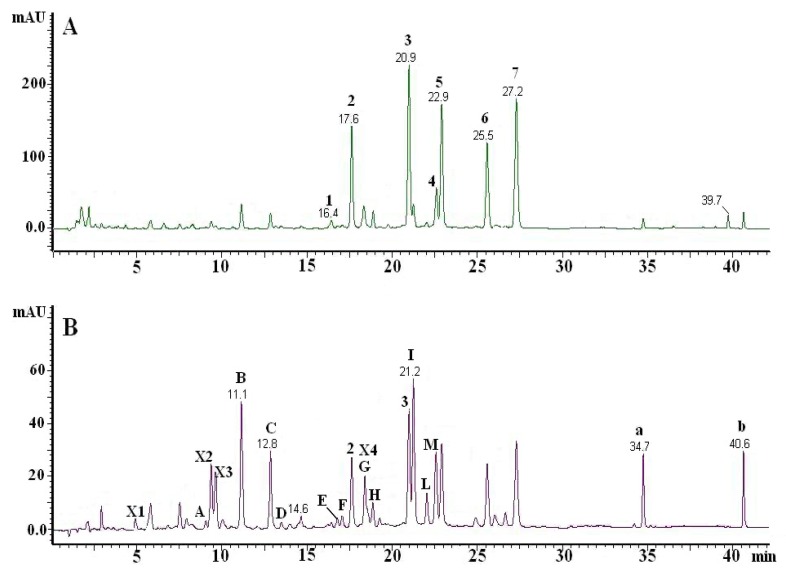
Typical chromatograms of bergamot juice at 282 nm (A) and 330 nm (B).

The analyzed BJ contained different classes of compounds like flavonoids, cinnamic acid derivatives and furanocoumarins, whose structures are given in Figure 1. Bergamot juice is a complex food matrix and the identity of its components can be correctly assessed by LC coupled with DAD and a tandem mass spectrometer. A good separation of flavonoids in BJ was achieved with a Luna(2) C_18_ narrow bore column with gradient elution. Figure 2 shows the chromatograms of a BJ sample at 282 nm (A) and 330 nm (B). The identity of peaks **2**, **3**, **5**, **E**, **I**, **M**, **a** and **b** was established by co-chromatography, “on-line” UV spectra comparison and molecular ion evaluation. Compounds **A-D** showed an UV spectrum suggesting a structure of a flavone-derivative and in MS^2^ they yielded typical ions product corresponding to (M-H)^-^, [(M-H)-18]^-^, [(M-H)-90]^-^, [(M-H)-120]^-^ and [(M-H)-210]^-^. The losses in the MS/MS coincided with those reported by Ferreres *et al*. [16] for di-C-symmetric hexosyl flavones, suggesting that peaks **A**-**D** were 6,8-di-C-glucosyl derivatives of luteolin, apigenin, chrysoeriol and diosmetin, respectively. 

**Figure 3:**
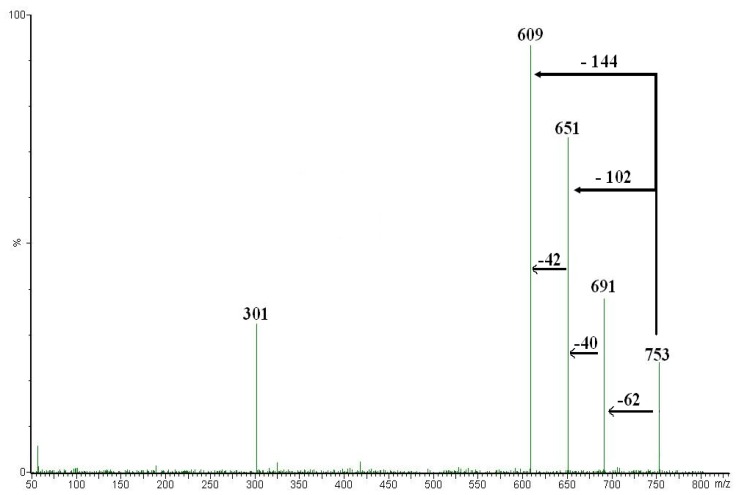
Fragmentation pattern of the peak **7** (MW 754) obtained at lower collision energy. The ions product with (m/z)^-^ 609 Da and (m/z)^-^ 301 Da correspond to hesperidin and hesperetin, respectively.

The MS/MS spectra of peaks **F**-**H** showed the presence of ions corresponding to (M-H)^-^, [(M-H)-90]^-^ and [(M-H)-120]^-^ and the absence of the [(M-H)-18]^-^ suggesting the presence of a single hexose moiety at the 8 position. Therefore, peaks **F**-**H** could be identified as 8-C-glucosyl derivatives of apigenin, chrysoeriol and diosmetin, respectively. Peak **1** showed an UV spectrum suggesting a structure of a flavanone-derivative and mass spectra yielded the same pseudomolecular ion with (m/z)^-^ 595 Da. At low collision energy values this ion gave an ion product with (m/z)- 285 Da, likely corresponding to the aglycone moiety. The mass data, combined with the UV and chromatographic behavior suggest that peak **1** was eriocitrin. Peaks **4**, **6** and **7** showed an UV spectrum suggesting a flavanone-derivative structure and their mass spectra showed pseudomolecular ions with (m/z)^-^ 739 Da, 723 Da and 753 Da, respectively. The MS-MS spectra of these parent ions displayed the presence of ions corresponding to [(M-H)-62]^-^, [(M-H)-102]^-^, [(M-H)-144]^-^ and (M-H)^-^. In positive mode peaks **4**, **6** and **7** gave the same ions corresponding to [(M+H)-146]^+^ and typical fragments with (m/z)^+^ 289 Da, 273 Da and 303 Da corresponding to eriodyctiol, naringenin and hesperetin, respectively. Moreover, the CID-MS/MS of the peaks **4**, **6** and **7** gave ions with (m/z)- 151 Da, which is a common fragment obtained from flavanone aglycones not substituted on the A ring. The retention time, UV behavior, negative and positive MS-MS spectra and the presence of neohesperidosides in BJ lead to assume that peak **4**, **6** and **7** could be neoeriocitrin, naringin and hesperidin esterified with two oxalic acid moieties, respectively. Figure 3 shows the fragmentation pattern of peak **7**. The presence of the ions product with (m/z)- 609 Da, corresponding to hesperidin, lead us to suppose that ions with (m/z)- 691 Da and 651 Da derive from the cleavage of oxalate moieties. 

Peaks **X1**, **X2** and **X3** showed an UV spectrum suggesting to the structure of 3,4-di-hydroxylated-cinnamic acid derivatives and displayed [M-H]^-^ ions at (m/z)^-^ 263 Da, 355 Da and 385 Da, respectively. Peak **X1** showed ions with (m/z)^-^ 221 Da [(M-H)-42]^-^, (m/z)^-^ 179 Da (caffeic acid) [(M-H])-84]^-^ and [m/z]^-^ 161 Da (caffeic acid-H_2_O) in its MS^2^ spectra. Based on the above observations peak **X1** was tentatively identified as caffeic acid esterified with two acetic acid moieties.

In the MS^2^ spectra of peak **X2** the main fragments were the ions with (m/z)^-^ 193 Da (ferulic acid) [(M-H)-162]^-^, (m/z)^-^ 175 [ferulic acid-H_2_O]^-^ and [m/z]^-^ 163 Da [(ferulic acid-OCH_3_)]^-^. Analogously, compound **X3** showed losses from the pseudomolecular ion [M-H]^-^ of 162 Da (hexosyl moiety) to give typical ions with (m/z)^-^ 223 Da (sinapic acid). In the MS^2^ spectra of peak **X3** ions with (m/z)^-^ 205 Da (sinapic acid-H_2_O) and (m/z)^-^ 163 Da [(sinapic acid-OCH_3_-OCH_3_)]^-^ were also present. Thus, it seems that peaks **X2** and **X3** were ferulic and sinapic acid esterified both with one hexosyl moiety, reasonably glucose. 

Peak **X4** yielded a UV spectrum with maxima at 250 nm and a shoulder at 275 nm, and pseudomolecular ions with (m/z)^-^ 397 Da. The MS^2^ analysis of this precursor gave ions with (m/z)-235 Da [(M-H)-162]^-^, which could correspond to the aglycone moiety. Thus, Peak **X4** could be considered the hexosyl derivative of an aglycone with molecular weight 236 Da.

Flavanone and flavone aglycones were not found in the analyzed BJ, while their O- and C-glycosides represented the most abundant constituents. The contents of flavonoids, furanocoumarins and cinnamic acid derivatives found in bergamot juice are reported in Table 1. The amount of flavonoids and furanocoumarins were determined using calibration curves obtained with standards, while for compounds **X1**, **X2-X3** caffeic and ferulic acid calibration curves were used, respectively. Their amounts were then normalized by the molecular mass ratios. The amount of flavonoids determined in this paper agrees with those reported in literature [17], but they are not in agreement with other studies indicating higher amounts of flavanones [14]. The discrepancy could be ascribed to different factors such as: BJ preparation procedure, BJ obtained from various cultivars, different maturation period or the use of unspecific chromatographic methods not able to separate neoeriocitrin, naringin and hesperidin from their di-oxalate derivatives. Regarding furanocoumarins, we found in BJ bergamottin amounts lower than that reported in literature [17].

**Table 1:** Content of flavanone-O-glycosides (**1-7**), flavone-C-glucosides (**A-H**), flavone-O- neohesperidosides (**I-M**), cinnamic acid derivatives (**X1-3**) and furanocoumarins (**a-b**) in BJ.

Peak	mg/L	Peak	mg/L
**1**	9.6±1.1	**F**	2.8±0.3
**2**	73.3±1.6	**G**	9.1±0.7
**3**	167.5±1.8	**H**	6.0±0.3
**4**	20.1±1.1	**I**	46.4±1.9
**5**	123.9±1.7	**L**	7.8±0.4
**6**	89.3±2.2	**M**	23.1±1.4
**7**	190.0±3.1	**a**	9.0±0.4
**A**	1.3±0.1	**b**	18.2±0.5
**B**	38.6±2.1	**X1**	1.6±0.2
**C**	25.8±1.3	**X2**	12.6±0.9
**D**	2.0±0.1	**X3**	12.1±0.8
**E**	2.1±0.1		

Regarding the validation method, calibration curves were constructed for each standard at five concentration levels and three independent determinations were performed at each concentration. The recovery of the extraction from spiked BJ samples was 97.2±3.1. The precision of the method was tested by both intra-day (n=5) and inter-day (5 days, n=5) reproducibility, and the coefficient of variation was below 3.3 %. Limit of quantization and limit of detection were 2 μg/ml and 1 μg/ml, respectively. 

## Conclusions

Bergamot juice contains several different compounds and LC-DAD-MS is the best choice for their separation, identification and quantization. Moreover, tandem mass spectrometry with collision induced dissociation allows structural identification, especially when standard compounds are not available.

## Experimental

### Chemicals

Naringin, narirutin, hesperidin, neohesperidin, neoeriocitrin, isovitexin, rhoifolin, neodiosmin, bergapten and bergamottin were from Extrasynthese (Genay, France). Caffeic and ferulic acid were from Sigma (St. Louis, MO, USA). Methanol, acetonitrile and formic acid were purchased from Merck (Darmstadt, Germany). Water was obtained from a MilliQ apparatus (Millipore, Milford, MA). The bergamot fruits were a gift of the “Consorzio del Bergamotto” (Reggio Calabria, I). 

### Method validation

The LC-DAD-MS method was validated for linearity, LOQ, LOD, accuracy, peak purity, precision and repeatability. Lower limit of quantization (LLOQ, *S*/*N* ratio of 8) and lower limit of detection (LLOD, *S*/*N* ratio of 3) were determined by serial dilution of standard solutions. Accuracy was evaluated by spiking three BJ sample with four amounts (2, 5, 10 and 20 μg/mL) of the mix standard compounds. Peak purity and identity were confirmed by LC-DAD-MS and LC-MS^2^ experiments. Precision (intra- and inter-day) of the assay was verified by analyzing BJ samples 3 times on five consecutive days. Repeatability was confirmed by evaluating standard deviations of the retention times and peaks area. 

### Sample preparation

Bergamot fruits (1 Kg) were peeled, squeezed and the obtained juice was mixed and apportioned in 20 mL containers, which were stored at -80°C. Ten mL were diluted 5-fold in methanol-formic acid 1% (90:10, v/v) and the resulting solution was sonicated for 10 min and then centrifuged at 1000 x g for 10 min. One aliquot of the supernatant was filtered through a Millipore 0.2 μm disk and 5 μL were injected into the LC system. 

### LC-DAD-MS/MS analysis

The chromatographic system was an Alliance mod. 2695 (Waters, Milford, MA) equipped with a mod. 2996 (Waters) photodiode array detector and a triple quadrupole mass spectrometer mod. Quattro micro (Micromass, Beverly, MA). A 3 μm C_18_ Luna (2) narrow bore column (150 x 2.0 mm, Phenomenex, Torrance, USA) was used for the separation, which was performed by means of a linear gradient elution (eluent A, 0.1% formic acid; eluent B, acetonitrile) at a flow rate of 250 μL/min. The gradient was as follows: from 10 to 25% B in 20 min, 25% B for 5 min, 25 to 50% B in 5 min, 50 to 95% B in 5 min and then 95% B for 15 min. The column was maintained at 30°C. Chromatographic data were acquired in the 200-450 nm range and were integrated at 282 nm (flavanones) and 330 nm (flavones, furanocoumarins and cinnamic acid derivatives). The mass spectrometer operated in positive and negative full-scan mode in the range 100-1000 Da. The capillary voltage was set to 3.0 kV, the cone voltage was 20V, the source temperature was 130°C, and the desolvating temperature was 350°C. All data were acquired by Masslink 4.0 software (Micromass) with the Quan-Optimize option for the fragmentation study. Calibration curves were obtained from narirutin, naringin, hesperidin, neohesperidin, rhoifolin, neodiosmin, neoeriocitrin, bergapten, bergamottin, caffeic and ferulic acid stock solutions prepared by dissolving 5 mg of standard powder in 5 mL methanol. They were measured in the range of 2-20 μg/mL.
